# A cucumber NAM domain transcription factor promotes pistil development in *Arabidopsis*

**DOI:** 10.1186/s43897-021-00013-w

**Published:** 2021-09-15

**Authors:** Jian Pan, Hai-Fan Wen, Wen-Hui Lin, Jun-Song Pan

**Affiliations:** 1 School of Agriculture and Biology, Shanghai Jiao Tong University, Shanghai, China; 2 School of Life Sciences and Biotechnology, The Joint International Research Laboratory of Metabolic & Developmental Sciences, Joint Center for Single Cell Biology, Shanghai Jiao Tong University, Shanghai, China

## Introduction

Sexual reproduction is a fundamentally important biological process in nature. Unisexual flowers provide a widely used system for studying plant sex organ differentiation. Cucumber (*C. sativus* L.) generates both unisexual and bisexual flowers, and the sex type is mainly controlled by several ethylene synthases, i.e., *CsACS2*, *CsACS1G*, *CsACS11*, and *CsACO2* (Boualem et al., 2008; Martin et al., 2009; Boualem et al., 2015; Chen et al., 2016). The unisexual flowers typically result from the selective suppression of one of the two genders in flower development stages 6–7 (Bai et al., 2004). Meanwhile, if the suppression is deactivated, caused by loss-of-function *CsACS2* (*CmACS7* in melon), functional stamens develop to make the flower bisexual (Li et al., 2009). The prior studies have demonstrated that an ethylene positive feedback regulation mediates by *CsACS2* and *CsERF31* is pivotal for cucumber female flower differentiation (Li et al., 2012; Pan et al., 2018). However, the molecular mechanism of how the ethylene signal promotes female organ development remains elusive. Here, we identified a cucumber putative NAM domain transcription factor, *CsNAC2*, that showed specific female organ expression. Ectopic expression of *CsNAC2* in *Arabidopsis* led to carpelloid stamens and decreased the ethylene accumulation, implying fine-tuned ethylene regulation post-sexual differentiation in cucumber. Our finding provides new clues to understand the regulatory mechanism of female organ differentiation.

## Results

**Fig. 1:**
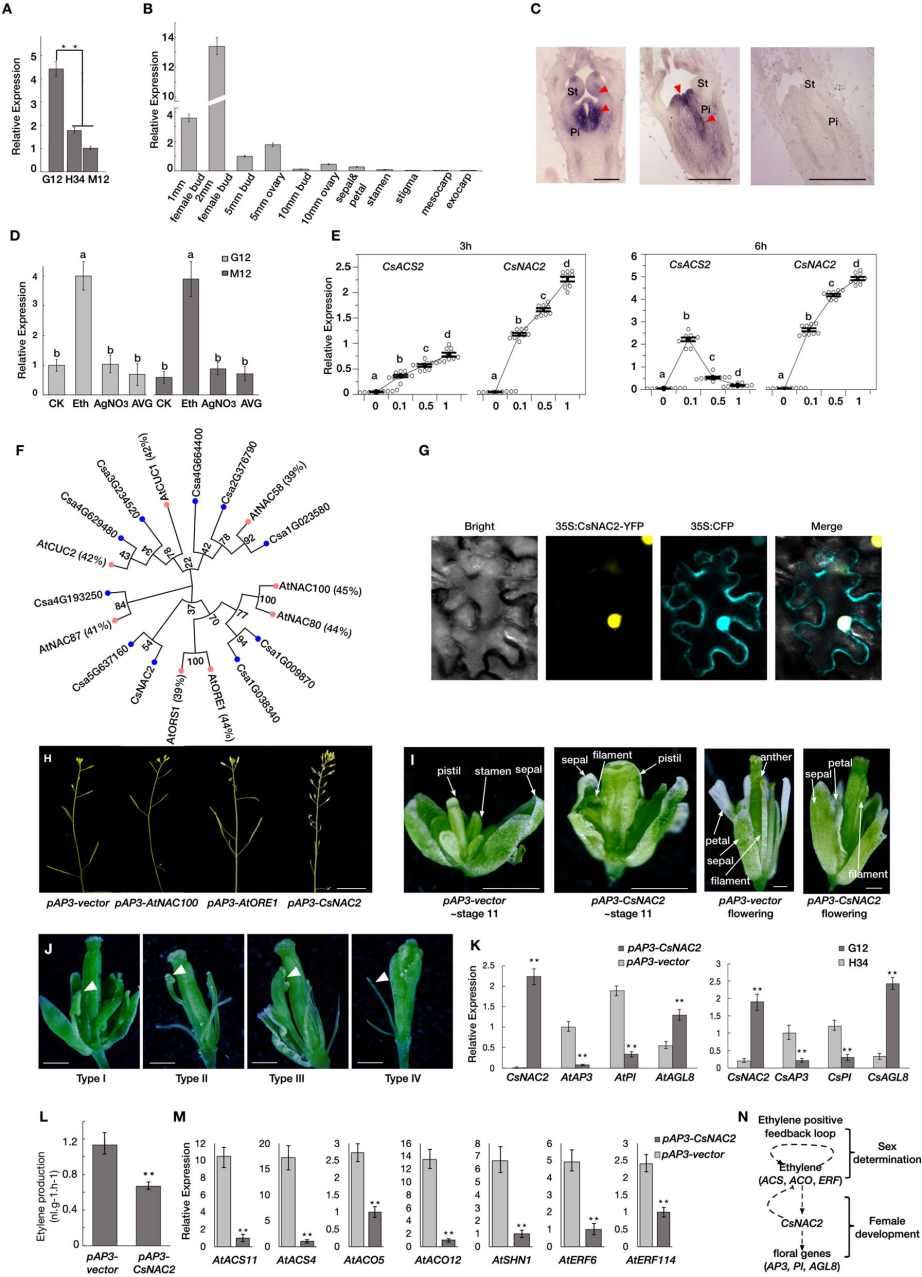
**A** Expression analysis of *CsNAC2* in 1 mm buds of different genotype cucumber lines. Gynoecious line, G12 (*CsACS1G*/*CsACS2*); monoecy line, M12 (*CsACS1*/*CsACS2*); and hemaphrodite line, H34 (*CsACS1G*/*csacs2*). Error bars represent the SD from three biological replicates, and asterisks indicate significant differences in different treatments (*t*-test, ** *p* value < 0.01). **B** Spatial-temporal expression patterns of *CsNAC2* in female development. Temporal expression patterns at different stages of female bud (1, 2, 5, and 10 mm buds were divided into 5 mm bud, 5 mm ovary, 10 mm bud, and 10-mm ovary, respectively) and spatial expression patterns of petal, stamen, stigma, mesocarp, and exocarp. Error bars represent the SD from three biological replicates. **C** In situ hybridization of *CsNAC2* in gynoecy cucumber buds. Hybridization with antisense-probe in stage 7 (left), stage 8–2 (middle). Red arrows indicate hybridization signal. Hybridization with sense-probe presents in right. St: stamen, Pi: pistil. Bar = 200 μm. **D** Expression patterns of *CsNAC2* in shoot apices of the chemical treatments and control cucumber lines. Eth: ethephon, AgNO3: silver nitrate, AVG: aminoethoxyvinyl-glycine. Error bars represent the SD from three biological replicates. Different letters (a, b) indicate significant differences (one-way ANOVA, *p* value < 0.01). **E** Expression level of *CsNAC2* and *CsACS2* in cucumber shoot apices of different concentrations treatment. Apical shoots of four-leaf stage seedlings were soaked in water containing 0, 0.1, 0.5, and 1.0 mM ethephon for 3 and 6 h, respectively. The circle indicates the expression of 10 biological replicates. The letters indicate the difference between each other *p* value < 0.01 in (a, b), (b, c) and (c, d) from *t*-test. **F** An unrooted phylogenetic tree of CsNAC2 from Arabidopsis and cucumber. The phylogenetic tree was constructed by the Neighbor-Joining method with 1000 bootstrap sampling. Bar represents patristic distances. **G** Subcellular localization of CsNAC2. Subcellular localization of CsNAC2-YFP fusion proteins in tobacco leaves. 35S:CFP indicates a marker for nuclear localization signal. **H** Phenotype result of *Arabidopsis* transgenic individual. Phenotypes of transgenic plants are T2 generation. All transgenic plants in different individual lines share the same phenotype, and only one line shows in result. **I** Floral organs phenotype of *Arabidopsis* transgenic individual. Flower bud phenotype of *pAP3-vector* and *pAP3-CsNAC2* in stage 11(left) and flowering time (right). Bar = 0.2 cm. **J** The four types phenotypes of *pAP3-CsNAC2* flower buds. Type I, normal looking but non-functional stamen, with papillar tissue on the tips. Type II, carpelloid stamen fuse with carpel. Type III, a carpelloid stamen not fuse with carpel. Type IV, no stamens and abnormal carpel. The white arrows indicate the abnormal floral organs. Bars = 0.2 cm. **K** Expression level of floral genes in transgenic *Arabidopsis* (left) and cucumber (right) plants. Error bars represent the SD from three biological replicates, and asterisks indicate significant differences in different treatments (*t*-test, ** *p* value < 0.01). **L** Ethylene production in *pAP3-CsNAC2* the transgenic *Arabidopsis* plants. Error bars represent the SD from four biological replicates, and asterisks indicate significant differences in different treatments (Student’s *t*-test, ** *p* value < 0.01). **M** Expression level of ethylene related genes in the transgenic *Arabidopsis.* Error bars represent the SD from three biological replicates, and asterisks indicate significant differences in different transgenic plants (*t*-test, ** *p* value < 0.01). **N** Model of *CsNAC2* functions during the development of female flowers in *Arabidopsis* and cucumber. The ethylene signal activated by the positive feedback loop induces the expression of *CsNAC2*. *CsNAC2* then promotes female organ development via activates floral genes in an unknown way, and represses the ethylene related genes to avoid an excessive ethylene effect in the flower bud

Having identified the ethylene positive feedback regulation in the cucumber female flower determination (Pan et al., 2018; Pan et al., 2021), we next studied how highly accumulated ethylene promoted female organ differentiation. Based on our previous transcriptomic analysis in the shoot apexes of different genotypes in cucumber (Pan et al., 2018), we found a NAM domain transcription factor, *CsNAC2*, which had a specific high expression in gynoecious line. In this study, we tested the mRNA expression level of *CsNAC2* in the flower buds (before stage 7, according to (Bai et al., 2004)) of different genotypes, as gynoecious line, G12 (*CsACS1G*/*CsACS2*); monoecy line, M12 (*CsACS1*/*CsACS2*); and hemaphrodite line, H34 (*CsACS1G*/*csacs2*). *CsNAC2* showed a more significant high expression in the female bud in G12 (Fig. 1A), suggesting its function might relate to female flower development. Although *CsNAC2* displayed a relatively higher expression in 1 mm (before stage 7) predetermined female bud, the highest expression is in the 2 mm (~ stage 8–2) female bud, which differs from most sex-determination genes in cucumber, like *CsACS2*, *CsACS11* and *CsACO2* (Fig. 1B) (Zhang et al., 2020). The higher expression in 2 mm female bud suggested that *CsNAC2* might promote female organ primordium differentiation after the sex determination. In situ hybridization further showed that the transcripts of *CsNAC2* were specifically accumulated in both stamen and pistil primordia in the predetermined 1 mm bud; then only in the pistil primordium of the 2 mm female flower bud (Fig. 1C).

Given that ethylene has been widely accepted as a critical regulator of cucumber female flower development, we further tested the effect of ethylene-related treatments on the expression of *CsNAC2* on the gynoecious and monoecious cucumber lines. AgNO_3_ and AVG are known to interfere with ethylene action, and their application promoted the conversion of female flowers into male flowers. The results showed that the expression of *CsNAC2* was not significantly changed in AgNO3 and AVG treatments. However, ethephon could induce the *CsNAC2* expression in both gynoecious and monoecious lines (Fig. 1D). Therefore, we further tested the expression level of *CsACS2*, as a marker of female flower initiation, in different concentration ethephon treatments. We found *CsACS2* was promoted under low level (0.1 mM) and was inhibited under high level (0.5 and 1 mM) (Fig. 1E), which was consistent with the prior study (Li et al., 2012). However, the expression pattern of *CsNAC2* was not followed that of *CsACS2*, and instead displayed a positive correlation with the ethylene concentration (Fig. 1E).

The result of phylogenetic analysis suggested that CsNAC2 has a relatively closer evolutionary relationship with AtNAC100 (45%), AtORE1 (44%), and AtNAC80 (44%) in amino acid sequence (Fig. 1F). Subcellular localization analysis indicated that CsNAC2 was localized in the nucleus, which suggested a putative transcription factor (Fig. 1G). To determine if *CsNAC2* has a role in regulation of female organ differentiation, we constructed transgenic *Arabidopsis* plants with the organ-specific expression of *CsNAC2* under the control of an *AtAP3* promoter, which specifically expressed in stamens. To verify the potentially distinguish function of *CsNAC2* in promoting female development, we used the *AtAP3* promoter drove *AtNAC100*, *AtORE1* (*ORESARA1*) and the empty vector as controls (Fig. 1H). The phenotype of the *pAP3-CsNAC2* transgenic *Arabidopsis* plants showed that stamen and petal development were significantly altered, resulting in male sterility (23 out of 24 individuals) (Fig. 1I). For every individual among the 23 transgenic plants, four types of stamens could be classified according to their morphology. Type I (~ 38%): normal-looking but non-functional stamen, with papillar tissue on the tips. Type II (~ 21%): carpelloid stamen partially fuse with carpel. Type III (~ 29%): a carpelloid stamen did not fuse with the carpel. Type IV (~ 12%): no stamens and abnormal carpel (Fig. 1J). We performed pollination tests to confirm whether *CsNAC2* expression affected the pistil development and found that the transgenic *Arabidopsis* plants could generate fertile offspring. The above results indicated that the ectopic expression of *CsNAC2* with *AP3* promoter promotes female organ differentiation, which is conserved with the condition in cucumber. The B gene, i.e., *AP3* and *PI*, are known as the ruler of stamen and petal development (Bowman et al., 1991). Thus, we tested the expression level of *AtAP3* and *AtPI* in *pAP3-CsNAC2 Arabidopsis* plants and found that both of them were significantly down-regulated. This is consistent with reduced expression of *CsAP3* and *CsPI* in gynoecious (G12) vs. hermaphrodite (H34) cucumber lines (Fig. 1K). Notably, *AtAGL8*, which controls the carpel development and fruit cell differentiation in *Arabidopsis* (Gu et al., 1998), showed two-folds upregulated in *pAP3-CsNAC2* plants, and its ortholog *CsAGL8* showed a similar upregulated expression in gynoecious cucumber line (G12 vs. H34 in Fig. 1K). The above results suggested that the regulatory mechanism of female organ promotion might be conserved in *Arabidopsis* and cucumber. Furthermore, we found CsNAC2 showed a DNA binding activation but cannot directly interact with the promoter of *AP3*, *PI*, or *AGL8*, via yeast one-hybrid and dual-luciferase assay (Supplemental Information Fig. S1).

Given the important role that ethylene plays in cucumber female flower development, we tested the ethylene productivity in the transgenic *Arabidopsis* flower buds. The results indicated ethylene production was significantly decreased in *pAP3-CsNAC2* (Fig. 1L), suggesting that the expression of *CsNAC2* suppressed the ethylene accumulation. We next investigated the expression level of several ethylene synthetases (ACSs and ACOs) and ethylene response factors (ERFs), and found that *AtACS11*, *AtACS4*, *AtACO5*, *AtACO12*, *AtSHN1*, *AtERF6,* and *AtERF114* were significantly downregulated in *pAP3-CsNAC2* plants (Fig. 1M). Thus, our combined data suggest that *CsNAC2* is downstream of ethylene signal, and it may function in repressing male organ development and inducing female organ development, and regulating ethylene production (Fig. 1N).

## Discussion

In the last decades, sufficient evidence has been reported to support ethylene promotes cucumber female flower development; however, we still gain little knowledge about the regulatory mechanism of how ethylene selectively promoting floral gene expression to produce the proper unisexual flower. In this study, we demonstrated the role of *CsNAC2* in responding to the upstream ethylene signal and promoting female organ development, meanwhile limiting the accumulation of ethylene produce (Fig. 1N). This result is consistent with the hypothesis that the amplified ethylene signal, which decides female initiation, should be limited to a certain level to avoid damaging organ primordium development (Li et al., 2012; Pan et al., 2018). Besides, our results imply that *CsNAC2* may act as a repressor to arrest the stamen development via indirectly down-regulating *AP3* and *PI* in both cucumber and *Arabidopsis* (Fig. 1K), meanwhile promotes the pistil development. Our data provided evidence to link *CsNAC2* expression pattern to female flower development in cucumber, and revealed a “carpelloid stamens” phenotype led by ectopic expressing *CsNAC2* in *Arabidopsis*, which could help to understand the regulatory mechanism downstream of ethylene signaling in female floral organ development.

### Supplementary Information

**Additional file 1.**

## Data Availability

All data generated or analyzed during this study are included in this published article.
