# Supplementary material for: A cucumber NAM domain transcription factor promotes pistil development in Arabidopsis
Source: Mol Hortic. 2021 Sep 15;1:10. doi: 10.1186/s43897-021-00013-w (PMC10515228; doi:10.1186/s43897-021-00013-w)
Supplement: Supplementary file 1 — Additional file 1. [file 43897_2021_13_MOESM1_ESM.docx]

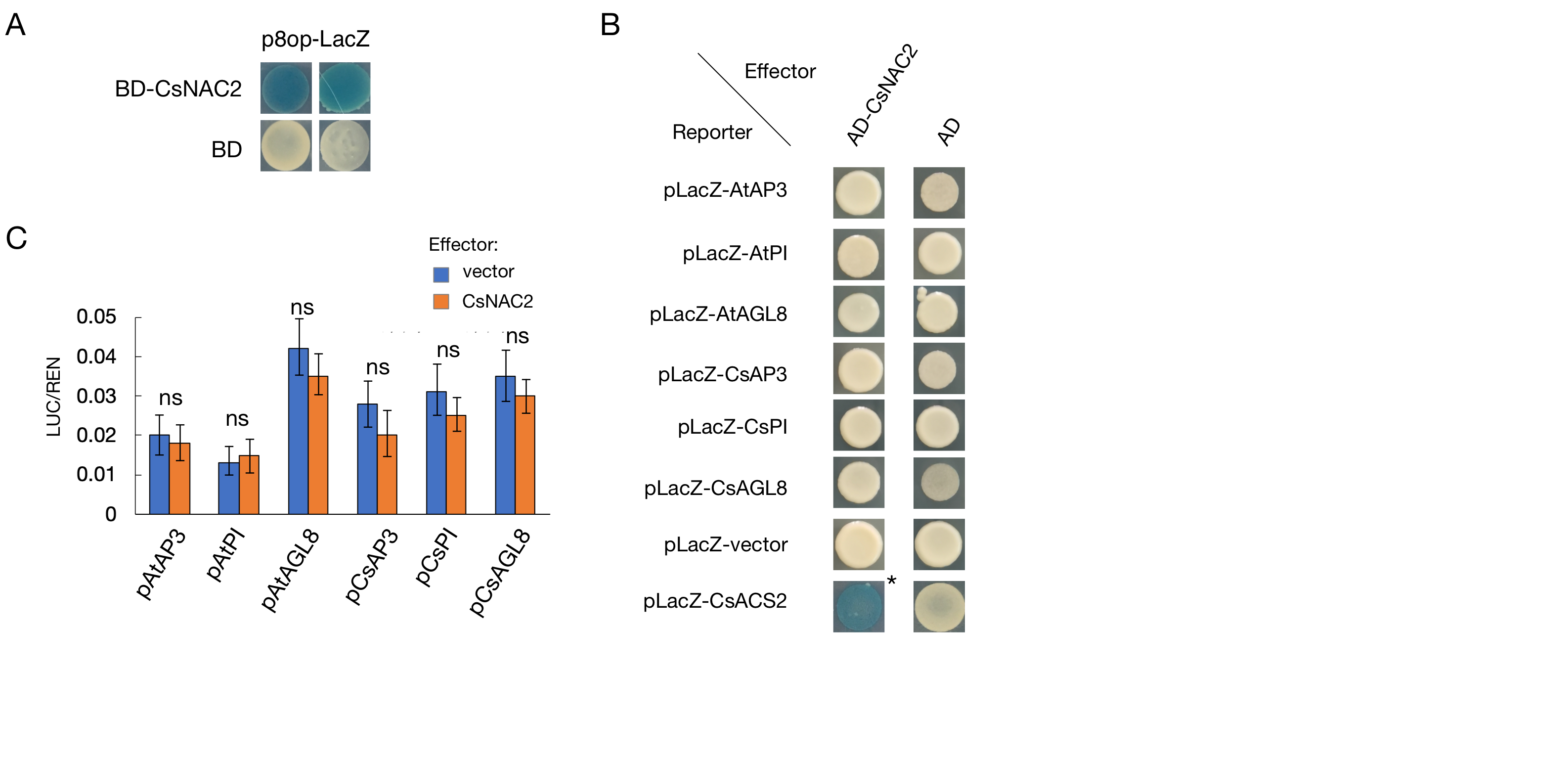


**Fig. S1 Interaction assays for *CsNAC2* and floral genes.** (**A**) Assays for transcriptional activation capacity of CsNAC2 protein. The transformed yeast strain with constructs of BD-CsNAC2 and p8op-LacZ were cultured on SD/-His/-Ura media. BD empty (pLexA) and p8op-LacZ were negative controls. (**B**) Yeast one-hybrid assay showing CsNAC2 cannot bind to any of these floral genes. The asterisk indicates the combination of AD-CsERF31 and pLacZ-CsACS2 which used as the positive control (Pan *et al.*, 2018). Blue color indicates expression of the LacZ reporter. The empty vectors pLacZ and pB42AD were used as negative control. (**C**) Schematic representation of the Dual-LUC assay reporter constructs expressing LUC under these floral genes promoter, which were inserted into pGreen as a reporter. 35S::CsNAC2 represented CsNAC2 coding sequence was inserted into pHB vector as an effector. Expression values were determined by calculating the ratio of LUC activity to REN activity (LUC/ REN). The error bars show ± SE of four biological replicates. Results were analyzed by Student’s *t*-test, “ns” indicates non-significant difference in *p* value>0.01.
